# PPARdelta in Affected Atopic Dermatitis and Psoriasis: A Possible Role in Metabolic Reprograming

**DOI:** 10.3390/ijms22147354

**Published:** 2021-07-08

**Authors:** Stefan Blunder, Petra Pavel, Deborah Minzaghi, Sandrine Dubrac

**Affiliations:** Epidermal Biology Laboratory, Department of Dermatology, Venereology and Allergology, Medical University of Innsbruck, Anichstraße 35, 6020 Innsbruck, Austria; stefan.blunder@i-med.ac.at (S.B.); petra.pavel2@gmail.com (P.P.); deborah.minzaghi@i-med.ac.at (D.M.)

**Keywords:** PPAR, atopic dermatitis, psoriasis, metabolic reprograming, glucose, fatty acids

## Abstract

Peroxisome proliferator-activated receptors (PPARs) are nuclear hormone receptors expressed in the skin. Three PPAR isotypes, α (NRC1C1), β or δ (NRC1C2) and γ (NRC1C3), have been identified. After activation through ligand binding, PPARs heterodimerize with the 9-cis-retinoic acid receptor (RXR), another nuclear hormone receptor, to bind to specific PPAR-responsive elements in regulatory regions of target genes mainly involved in organogenesis, cell proliferation, cell differentiation, inflammation and metabolism of lipids or carbohydrates. Endogenous PPAR ligands are fatty acids and fatty acid metabolites. In past years, much emphasis has been given to PPARα and γ in skin diseases. PPARβ/δ is the least studied PPAR family member in the skin despite its key role in several important pathways regulating inflammation, keratinocyte proliferation and differentiation, metabolism and the oxidative stress response. This review focuses on the role of PPARβ/δ in keratinocytes and its involvement in psoriasis and atopic dermatitis. Moreover, the relevance of targeting PPARβ/δ to alleviate skin inflammation is discussed.

## 1. PPARdelta: The Least Studied PPAR Isoform

Peroxisome proliferator-activated receptors (PPARs) are transcription factors belonging to nuclear hormone receptor superfamily. Three PPAR isotypes, α (NRC1C1), β or δ (NRC1C2) and γ (NRC1C3), have been identified in mammals (henceforth, we refer to the β/δ isoform simply as PPARδ). After activation through ligand binding, PPARs heterodimerize with the 9-cis-retinoic acid receptor (RXR), another nuclear hormone receptor, to bind to specific PPAR-responsive elements in regulatory regions of target genes, mainly involved in organogenesis, cell proliferation, cell differentiation, inflammation and metabolism of lipids or carbohydrates. Endogenous PPAR ligands are fatty acids and fatty acid metabolites.

PPARδ is ubiquitously expressed in murine tissues with highest expression in liver, muscle, adipose tissue, placenta, small intestine and skin. PPARδ is expressed twofold, 10-fold and 30-fold more in mouse keratinocytes (KCs) compared to mouse liver, quadriceps muscle and thymus, respectively. In most tissues, PPARδ localizes to the nuclear fraction of cells and is hardly detectable in the cytoplasm [1]. In humans, PPARδ mRNA and protein are highly abundant in the thyroid gland and placenta whereas high amounts of mRNA and moderate amounts of protein are detected in the cerebral cortex, skin and esophagus. Of note, inconsistency between protein and RNA levels of PPARδ has been observed in many human tissues and cell types (https://www.proteinatlas.org/ENSG00000112033-PPARD/tissue, accessed on 7 July 2021). There are five human and mouse PPARδ isoforms generated by alternative splicing, which is a mechanism potentially involved in PPARδ regulation, as some PPARδ splice isoforms exhibit reduced translation efficiency [2,3].

The ligand-binding pockets of PPARs have a distinct three-armed T shape, which allows not only straight fatty acids to bind them, but also ligands with multiple branches such as phospholipids and synthetic fibrates. The ligand-binding pocket of PPARδ is smaller than that of PPARγ or PPARα, which limits the binding of large ligands when compared to the other two PPAR isoforms [4]. PPARδ is activated by several endogenous ligands including certain long chain fatty acids (regardless of saturation status), dihomo-γ-linolenic acid, eicosapentaenoic acid, 15(S)-hydroxyeicosatetraenoic acid (HETE), and arachidonic acid, with affinities in the low micromolar range (Table 1). Supraphysiological doses of 8(S)-, 12(S)-, 12(R)-, and 15(S)-HETE efficiently activate PPARδ. 13(S)-hydroxyoctadecadienoic acid (HODE) is considered as weak PPARδ activator [5,6]. Controversial results have been found for prostacyclin (PGI2) and all-trans retinoic acid [7,8]. It has also been reported that 4-hydroxynonenal (4-HNE) and 4-hydroxydodecadienal (4-HDDE), the peroxidation products of polyunsaturated fatty acids, can activate PPARδ, although the mechanism remains unknown [9,10]. Synthetic PPARδ ligands include GW501516, GW0742 and L165041, which preferentially activate PPARδ as compared to PPARα or PPARγ [6]. Recently, 27 new synthetic PPARδ agonists (13 with low nanomolar EC_50_ values) have been discovered [11]. However, it is important to stress that preferential ligand does not mean exclusive ligand and that supraphysiological doses of any of the PPARδ ligands will activate other PPAR isoforms, and the same is true for all PPAR isoforms. For example, bezafibrate, which is known as a PPARα ligand, activates all three PPARs at concentrations ranging from 55 to 110 μM [12]. In the absence of ligand binding, the heterodimer PPARδ-RXR is associated with corepressors and histone deacetylases (HDACs), which inhibit its transcriptional activity. After ligand binding, PPARδ undergoes conformational changes that induce the release of the corepressors and allow it to bind coactivators [7].

The transcriptional activity of PPARδ is modulated by several factors, which are not well characterized but include post-translational modifications such as phosphorylation. Epidermal growth factor receptor (EGFR) has been recently shown to induce PPARδ phosphorylation at Y108 in response to epidermal growth factor (EGF) [13]. Although PPARδ contains several putative phosphorylation sites (Y108, T252, T253, T256), (https://www.phosphosite.org/proteinAction.action?id=24004&showAllSites=true (accessed on 9 May 2021)) [14], little is known about phosphoregulation of PPARδ, in contrast to PPARα and PPARγ. Both cyclic adenosine monophosphate (cAMP) and protein kinase A (PKA) activators increase the ligand-activated and basal activity of PPARδ and could be upstream signals that commit PPARδ to the regulation of glucose and lipid metabolism [14]. In contrast, PPARδ can also be sumoylated at K104, which inhibits its activity [14]. Desumoylation of PPARδ by small ubiquitin-like modifier (SUMO)-specific protease 2 (SENP2) promotes the transcriptional activity of PPARδ, which, in turn, upregulates fatty acid oxidation by enhancing the expression of long-chain-fatty-acid–CoA ligase 1 (ACSL1), carnitine palmitoyltransferase Ib (CPT1b) and mitochondrial uncoupling protein 3 (UCP3) in muscles of mice fed a high fat diet [15]. Moreover, PPARδ contains several ubiquitylation sites, which suggests a potential role of ubiquitin–proteosome degradation in the regulation of its cellular turnover (https://www.phosphosite.org/proteinAction.action?id=24004&showAllSites=true (accessed on 9 May 2021)). Degradation of PPARδ via the proteasome might prevent its accumulation in the nucleus and thereby moderate its cellular activity [16]. In line with this, overexpression of PPARδ in fibroblasts leads to its polyubiquitylation and rapid degradation, a process partially prevented by exposure to the PPARδ synthetic ligand GW501516 [17].

PPARs can also engage in transrepression of other transcription factors. Although transrepression between nuclear factor kappa-light-chain-enhancer of activated B cells (NF-κB), activator protein 1 (AP-1), CCAAT-enhancer-binding protein (C/EBP), signal transducer and activator of transcription (STAT) and nuclear factor of activated T-cells (NF-AT) has been well characterized for PPARα and PPARγ, little is known about transrepression in the context of PPARδ [18,19]. L-165041 is a PPARδ ligand that is less potent and selective than GW501516, yet it promotes the binding of PPARδ to the p65 subunit of NF-κB exerting anti-inflammatory effects [5,20]. Moreover, in the absence of ligand, PPARδ binds directly to the transcription factor B-cell lymphoma 6 (BCL-6), leading to increased expression of proinflammatory cytokines. Indeed, BCL-6 is a transcription factor repressing the expression of various inflammatory genes via direct binding to their promoters or via inhibition of the transcription of nucleotide-binding oligomerization domain-like receptor (NOD)-like receptor family pyrin domain containing 3 (NLRP3) [21,22]. Binding of PPARδ to an agonist disrupts the PPARδ-BCL-6 complex, thus reversing the transcriptional repression of inflammatory genes [23]. Thus, ligand binding to PPARδ alleviates inflammation by enhancing its binding to NF-kB, hence neutralizing the transcriptional activity of NF-kB and/or the release of the anti-inflammatory transcription factor BCL-6. However, PPARδ has also been shown to bind to the N-terminal part of p65 in the absence of exogenous ligand [5]. Therefore, the pro- vs. anti-inflammatory role of PPARδ might be context- and ligand-dependent. Moreover, conformational changes experienced by PPARδ after ligand binding might potentially strengthen or weaken the affinity of PPARδ to p65; however, this has not been studied to date.

Although there is likely a set of core effects and target genes of PPARδ common to all cell types and organs, PPARδ has also been shown to exert tissue-specific functions. Moreover, some target genes differ between rodents and humans. Canonical PPARδ target genes are mainly related to lipid metabolism in all cell types [6,19,24,25,26]. This includes genes involved in fatty acid oxidation (very long-chain specific acyl-CoA dehydrogenase, mitochondrial (*ACADVL*), acyl-CoA oxidase 1 (*ACOX1*), acetyl-CoA acyltransferase 2 (*ACAA2*), catalase (*CAT*), enoyl-CoA hydratase 1 (*ECH1*), pyruvate dehydrogenase kinase 4 (*PDK4*), solute carrier family 25 member 20 (*SLC25A20*), Niemann-Pick C1-like protein 1 (*NPC1L1*), *thiolase B*, *CPT1A*)) or other aspects of lipid metabolism (angiopoietin Like 4 (*ANGPTL4*), fatty acid binding proteins 3-5 (*FABP3-5*), perilipin 2 (*PLIN2*), adipocyte protein 2 (*aP2*)). Other PPARδ target genes exert non-metabolic functions and are involved in immune regulation, such as *CD300A*, *CD52*, LDL receptor related protein 5 (*LRP5*), *NLRC4* and phosphatase and actin regulator 1 (*PHACTR1*) [27]. In muscles, PPARδ controls (i) the entry of long chain fatty acids into cells via *SLC27A1*, *SLC27A3* and *CD36*; (ii) their subsequent activation by forming acyl-CoA via *ACSL3*, *ACSL4*, and acyl-CoA synthetases short chain family member 1 and 2 (*ACSS1-2*); (iii) mitochondrial β-oxidation via *CPT1A*, *CPT1B*, *SLC25A20*, *ACADVL*, and *ACADL;* (iv) peroxisomal β-oxidation via *ACOX1* [28]. In human macrophages, PPARδ regulates the expression of genes involved in lipid metabolism but also electron-transfer-flavoprotein, beta subunit (*ETFB*), electron transfer flavoprotein-ubiquinone oxidoreductase (*ETFDH*) and iron-sulfur cluster assembly 1 (*ISCA1*), which play important roles in electron transfer and iron-sulfur complex assembly and in the immune response via upregulation of *CD1D*, *CD36*, *CD52*, *CD300A*, *LRP5*, *NLRC4* and *PHACTR1* and downregulation of *CCL8*, *CCL13*, *CXCL1*, *IL10*, *IL8* and *TNFA* [27].

The expression of *PPARD* is regulated by various cytokines, hormones, lipid metabolites and other transcription factors. The *PPARD* promoter region contains a vitamin D receptor (VDR) response element [29,30]. Thus, it is likely that there is cross-talk between VDR and the PPARδ pathway, but his has not been investigated in detail despite being of potential pathophysiological interest. AP-1, a transcription factor involved in the inflammatory response, and especially junB, both increase *PPARD* expression [31]. AP-1 mediates the effects of TNF-α, phorbol 12-myristate 13-acetate (TPA) and ceramides on *PPARD/Ppard* expression [32]. Tan et al., in a seminal work, showed that TNF-α promotes the synthesis of ceramides via sphingomyelin hydrolysis, which ultimately activates AP-1 via the mitogen-activated protein kinase kinase kinase 1 (MEKK1) and stress-activated protein kinases (SAPK)/Jun amino-terminal kinases (JNK)/p38 mitogen-activated protein kinases (p38MAPK) pathway [32]. Previous work also showed that *PPARD* can be upregulated by T3-thyroid receptor (TR) [33]. The metabolic regulation of PPARδ has been reviewed elsewhere [7].

## 2. Metabolic Features of Keratinocytes in Normal Skin

Data on metabolic pathway predominating in keratinocytes is still a controversial topic. Old literature suggests that, to generate ATP, KCs are predominantly committed to glycolysis in the presence of glucose or to mitochondrial respiration in its absence [34]. In suprabasal KCs, limited access to glucose from the dermal vasculature is believed to promote mitochondrial respiration and oxidation of lipids, in contrast to basal KCs, which preferentially use glucose as their main energy substrate [34,35,36]. In line with this, GLUT1 is the main GLUT isoform in the epidermis and is abundantly expressed in the basal layer, although residual expression can be found in suprabasal layers [37,38,39]. Recent work showed that decreased glycolysis via inhibition of glucose uptake in KCs promoted cell differentiation, suggesting a major role of glycolysis in KC fate [40]. However, another work proposes a predominating role of mitochondrial-derived ROS in basal KCs as a signal to induce differentiation [41]. This is in line with a recent report showing that NIX, a transcription factor located in mitochondria, controls mitophagy and, in turn, KC differentiation, hence emphasizing the role of mitochondria in KC fate [42]. Thus, further work is required to clarify the relative contribution of glycolysis versus oxidative phosphorylation (OXPHOS) in the control of homeostatic processes in the epidermis.

## 3. PPARdelta in Psoriasis and Atopic Dermatitis

Atopic dermatitis and psoriasis are two chronic and pruritic inflammatory skin diseases exhibiting pathophysiological commonalities, including impaired epidermal barrier function, immune hyper-responsiveness, and local and systemic symptoms modulated by environmental factors such as the skin microbiome and stress. Moreover, both diseases are associated with a major genetic risk factor, i.e., Filaggrin (*FLG*) loss-of-function mutations in atopic dermatitis and the HLA-Cw0602 allele in psoriasis vulgaris [43,44]. Furthermore, in both atopic dermatitis and psoriasis patients, nonlesional and lesional skin coexists, but the mechanism of transition from the non-affected to the affected condition remains unclear. Atopic dermatitis is one of the most common inflammatory skin diseases worldwide and characterized by skin features such as erythematous and papulovesicular eruptions with oozing, crusting and pruritus as well as associated systemic signs such as food allergies, allergic asthma and rhinitis, anxiety and sleep disorders. At the cellular level, atopic dermatitis is characterized by (a) the complex interplay between impaired epidermal barrier function owing to altered lipid composition of the stratum corneum lipid matrix i.e., a reduction in the chain length of structural lipids (fatty acids and ceramides), (b) a complex Th2-driven inflammation, (c) skin infiltration by eosinophils, basophils and inflammatory dendritic cells, and (d) an altered skin microbiota [43,45,46,47,48,49,50,51,52]. In psoriasis vulgaris, genetic risk factors predominantly affect innate immunity, and to some extent adaptive immunity (IL12p/IL-23R axis, Th1, Th17 cells). Similarly to atopic dermatitis, skin immunological abnormalities in psoriasis are complex and associated with comorbidities (e.g., arthritis and cardiovascular manifestations), pointing to a systemic immune hyper-responsiveness [44,50,53,54,55,56].

PPARδ is expressed in all skin cell types, including KCs, fibroblasts, sebocytes, hair follicle cells, melanocytes and Langerhans cells [19,57,58,59]. PPARδ is the predominant isoform in human KCs and is expressed throughout all epidermal layers [32,60]. Activation of PPARδ with synthetic ligands promotes the expression of human KC differentiation markers such as involucrin (*INV*) and transglutaminase 1 (*TGM1*) [60]. Although there is consensus on the pro-differentiative effects of PPARδ ligands and PPARδ activation in KCs, the effects on KC proliferation are more controversial, with studies showing reduced [60] or enhanced [31] KC proliferation after treatment with the PPARδ ligand L-165041 or GW-501516. Treatment of human KCs with L-165041 gave opposite outcomes in two distinct studies [31,60]. Yet, the use of different treatment regimens of L-165041, i.e., 0.05 μM for 3 days [60] and 1 μM for 7 days [31], might have been responsible for these divergent results, for example by inducing the recruitment of different cofactors and thus engaging PPARδ in different metabolic pathways. Moreover, the direct effects of ligands should not be underestimated because the use of PPARδ siRNA to test the requirement for PPARδ in the cellular response was not carried out in either studies [31,60]. In line with this, L-165041 can activate other PPAR isoforms, i.e., PPARα, PPARγ1 and PPARγ2 at doses as low as 0.05 μM [60]. This underscores that PPAR ligands can exert receptor-independent effects, that metabolic effects might vary with ligand concentrations (e.g., U- or bell-curves), and that the relative contribution of other PPAR isoforms after treatment with ligands might significantly influence experimental results, hence stressing the need for cautious interpretation of data [46]. Human KCs infected with a lentivirus containing an RNAi sequence directed toward PPARδ displayed reduced proliferative capacity, suggesting that PPARδ promotes, rather than dampens, proliferation of human KCs [31]. However, it is also possible that PPARδ exerts both proliferative and differentiative functions according to the cellular context, i.e., basal cells (early KCs, progenitor and stem cells) or suprabasal cells (differentiated cells). As in other cell types, PPARδ is likely a master regulator of fatty acid metabolism in KCs by increasing the uptake of long-chain fatty acids via upregulation of CD36 and fatty acid β-oxidation [60] (Table 2). However, the role of PPARδ in epidermal lipid and glucose metabolism remains under-investigated. Interestingly, the PPARδ target genes in KCs are not identical to those in other organs and cell types (Table 2), suggesting PPARδ has specific cellular functions in the epidermis.

The *PPARD*/*Ppard* gene is upregulated in lesional skin of patients with psoriasis vulgaris [5,31,61,62,63,64,65] and of mouse models of psoriasis [63,64]. However, although *PPARD* has been identified as a putative pathogenic gene in psoriasis [65], variants at the *PPARD* genomic locus have not been associated with psoriasis. In psoriatic plaques, PPARδ accumulates in KC nuclei in all epidermal layers [5]; however, subcellularly, PPARδ is found both in the cytoplasm and nucleus of KCs in the basal layer and in the stratum spinosum, whereas it is strictly found in nuclei in KCs in the stratum granulosum [5,64]. This suggests that PPARδ is constitutively activated by endogenous ligands in granular KCs of the epidermis in patients with psoriatic lesions [64]. Accordingly, endogenous PPARδ ligands can be produced in psoriatic lesions from the oxidation of arachidonic acid via ALOX8 (mouse) or ALOX12 (mouse and human) [64,66], two enzymes located in the stratum granulosum [66,67,68]. FABP5 is a fatty acid-binding protein expressed in the epidermis and has been shown to deliver endogenous lipid ligands to PPARδ in KC nuclei and to be a PPARδ target gene [69]. The expression of FABP5 parallels that of PPARδ at both the mRNA and protein levels in psoriasis [5,63]. Thus, in the suprabasal epidermis of psoriatic lesions, it is likely that PPARδ is constitutively activated by endogenous ligands such as arachidonic acid or its derivatives (eicosanoids), which are transported by FABP5 to the nucleus of granular KCs to promote PPARδ–mediated KC terminal differentiation and lipid β-oxidation. Specific overexpression and activation of human PPARδ in suprabasal mouse epidermis has been achieved by generating transgenic mice expressing a Cyp1A1-driven expression of human *PPARD* in KCs followed by topical treatment with the PPARδ agonist GW501516 [62]. Interestingly, these mice developed psoriasis-like inflammation associated with an increased Th17 immune response [62]. In this model, sustained activation of the STAT3 pathway is critically involved in the development of psoriasis-like disease [62]. The constitutive activation of PPARδ in suprabasal epidermis not only promotes terminal KC differentiation but also the production, in KCs, of IL-36 and the pleiotropic pro-inflammatory cytokine IL-1β. The latter can contribute to the activation of skin dendritic cells, which can in turn, skew naïve T cells toward a Th17 phenotype [62]. Moreover, suprabasal mouse KCs overexpressing the constitutively activated human PPARδ probably secrete soluble factors able to trigger the proliferation of basal KCs [62]. In addition, in psoriatic plaques, some PPARδ localize to nuclei in basal KCs to potentially sustain KC proliferation [5,64]. In line with this, previous work suggested that upregulation of PPARδ in the epidermis of psoriatic lesions might contribute to KC hyperproliferation via the upregulation of heparin-binding EGF-like growth factor (HB-EGF) at the mRNA and protein levels [31]. HB-EGF is a ligand that activates EGFR and is expressed in the basal layer of the epidermis, where it has been shown to accelerate wound healing [70]. This might be relevant for psoriasis because disease flares can be induced by physical trauma (the isomorphic or Koebner phenomenon) among other causes. Pioneering work on the pathogenesis of psoriasis showed increased levels of antimicrobial peptides in psoriatic skin breaks the innate tolerance to self-DNA which ultimately drives autoimmunity [71]. Moreover, human genomic DNA fragments enhance *TNFA* and *HBEGF* expression as well as KC proliferation, hence mimicking the KC phenotype in psoriatic skin lesions [72]. Thus, we can speculate that PPARδ in the basal epidermis of psoriatic plaques sustains KC proliferation via mechanisms involving HB-EGF. NF-kB has been shown to inhibit PPARδ-dependent transactivation. However, in lesional psoriasis, p65 NF-kB is sequestered in the cytoplasm of basal KCs, which might allow PPARδ to exert its transcriptional regulation on various genes, including those involved in KC proliferation [5].

PPARδ is upregulated in the epidermis of lesional atopic dermatitis when compared to non-lesional skin but to a lesser extent than in psoriatic lesions [31]. The expression of *FABP5* parallels that of PPARδ in psoriasis and atopic dermatitis [31,73]. Notably, the expression of *Ppard* and *Fabp5* is markedly increased in the epidermis of mouse models of lesional atopic dermatitis [38,74]. Similar to psoriasis, FABP5 is mainly localized to the nuclei of suprabasal KCs, suggesting efficient local generation of PPARδ ligands to sustain the activation of PPARδ [38]. Interestingly, the amounts of arachidonic acid, PGF2α and 5-HETE (PPARδ endogenous ligands) are increased in lesional skin of atopic dermatitis patients when compared to healthy skin [75]. The increased cleavage of membrane phospholipids via cPLA2 in the stratum granulosum can significantly contribute to the accumulation of arachidonic acid and its derivatives in lesional atopic dermatitis skin as well as in psoriatic lesions [76,77,78]. The role of PPARδ has been less investigated in atopic dermatitis than in psoriasis. However, in both diseases, PPARδ might induce KC hyperproliferation, enhance differentiation and contribute to inflammatory processes.

However, PPARδ can also be envisaged as a key regulator of metabolism, especially in the metabolic shift toward anaerobic glycolysis that has been recently evidenced in psoriatic and atopic lesions [38,79,80]. The production of lactate is largely increased in the epidermis of flaky tail mice and mice treated with MC903, two mouse models of lesional atopic dermatitis [38] and of mice treated with imiquimod, a mouse model of psoriasis [81]. Interestingly, the PPARδ ligand GW610742, when orally administered to *ob/ob* mice, induces lactate accumulation in the liver [82]. Indeed, PPARδ has been shown to regulate the expression of key enzymes involved in glucose metabolism, including in KCs (Table 2) [83,84,85]. PPARδ can promote anaerobic glycolysis by upregulating PDK, an enzyme that inactivates pyruvate dehydrogenase (PDH) via phosphorylation. PDH is the rate-limiting enzyme involved in pyruvate uptake in mitochondria, which ultimately favors oxidative phosphorylation [86]. Thus, inactivation of PDH by PPARδ-induced PDK inhibits pyruvate uptake in mitochondria, which, in turn, promotes anaerobic glycolysis [87]. In the epidermis of flaky tail mice, there is a shift toward anaerobic glycolysis associated with an enhanced PPARδ pathway including increased PDK1. In line with this, mitochondrial function is not enhanced in the epidermis of flaky tail mice despite a dramatic need for energy to sustain forced KC proliferation and to dampen inflammation [38]. These results are in line with previous work showing that PPARδ antagonism favors mitochondrial function [88].

PPARδ promotes β-oxidation of fatty acids in all cell types, including KCs (Table 2) [85,89,90]. In flaky tail mice, peroxisomal β-oxidation is upregulated when compared to that of healthy mice, with marked increases in the mRNA, protein and activity levels of ACOX1 [38], a well-known PPARδ downstream target [89,90]. This profile has been observed in another mouse model of lesional atopic dermatitis, i.e., mice topically treated with MC903 [38]. This treatment is associated with decreased proportions of very-long chain fatty acids and ceramides, especially with 24 and 26 carbons [38], as observed in the epidermis of patients with lesional atopic dermatitis [91]. Interestingly, C24 and C26 fatty acids are exclusively oxidized in peroxisomes via ACOX1 [92,93]. Thus, the upregulation of PPARδ in the epidermis of patients with lesional atopic dermatitis might promote peroxisomal β-oxidation of very- and ultra-long-chain fatty acids and ceramides, hence significantly contributing to disease pathogenesis. Indeed, the efficacy of the stratum corneum barrier depends, to a large part, on the lipid composition of the lipid matrix surrounding the corneocytes, which consists of more than 50% fatty acids with 24 and 26 carbons. Interestingly, the proportion of very-long-chain ceramides is also decreased in the epidermis of psoriatic lesions [94] and is associated with increased ACOX1 [38] and PPARδ (see above), thus corroborating the key role of the PPARδ pathway in lipid abnormalities in both lesional atopic dermatitis and psoriasis. In contrast to lesional AD [38], mitochondrial β-oxidation might be increased in psoriasis as suggested by previous work [46] and might further contribute to lipid abnormities.

PPARδ has been shown to be involved in wound healing [95], which might demonstrate relevance in both psoriasis and atopic dermatitis. Indeed, both diseases are characterized by epidermal barrier impairment that can be considered as superficial wounds. In wounded epidermis, PPARδ inhibits KC apoptosis via activation of the phosphoinositide-3-kinase (PI3K)/PKBα/Akt1 pathway and promotes the re-epithelialization of the skin by enhancing KC adhesion and migration [95]. The upstream signal promoting the expression and activation of PPARδ in wounded epidermis is believed to be the accompanying low-grade inflammation, i.e., increased IL-1β and TNF-α, which promotes the synthesis of lipids and the release of bioactive lipids activating PPARδ [95]. In human epidermal equivalents (HEEs) topically treated with sodium dodecyl sulfate (SDS) to inflict epidermal barrier impairment, *PPARD* expression was upregulated at 24 h but not at 6 h post-treatment [96]. This upregulation of *PPARD* requires a rather strong epidermal barrier impairment because a milder epidermal barrier impairment induced by topical treatment of HEEs with acetone, did not result in *PPARD* upregulation [96]. Furthermore, the relatively late upregulation of *PPARD* suggests that it requires the prior synthesis of modulating factors such as lipids and/or cytokines. In line with this, IL-1β but not TNF-α, which are both upregulated after epidermal barrier impairment, is capable of upregulating *PPARD* in KCs [96]. Moreover, epidermal barrier impairment leads to excessive transepidermal water loss, a phenomenon described in both lesional atopic dermatitis and psoriatic plaques as well as in wounded skin. It is thus possible to speculate that, in this context, IL-1β upregulates PPARδ signaling including anaerobic glycolysis via PDK1 and peroxisomal β-oxidation via ACOX1 [38]. In line with these data, placement of occlusive dressing onto the skin of flaky tail mice to reduce transepidermal water loss was found to downregulate ACOX1 [38]. Another candidate upstream of PPARδ in the basal epidermis might be silent mating type information regulation 2 homolog 1 (SIRT1), which is known to promote wound healing [97,98] and enhance PPARδ transcriptional activity [99]. Thus, the chronic epidermal barrier impairment observed in lesional atopic dermatitis and psoriasis might lead to the constitutive activation of a sequential cellular compensatory response aimed at repairing the barrier; this could include upregulation of SIRT1 and production of IL-1β and subsequent release of bioactive lipids to activate PPARδ. This might ultimately lead to uncontrolled inflammation and disruption of epidermal homeostasis. Indeed, PPARδ has been shown to upregulate several genes involved in KC differentiation (e.g., *INV*, *S100A8*, *S100A9*, *TGM3*, *TGM1*) and proliferation (e.g., *HB-EGF*, *IL1B*, *IL17*, *IL22*) and the inflammatory response (e.g., *IL1B*, *IL18*, *IL1A*, *IL1RA*, *IL1F, IL17, IL22*) (Table 2).

KC hyper-proliferation, accelerated differentiation and the inflammatory response in psoriatic and atopic lesions require energy that might be provided by enhanced peroxisomal fatty acid β-oxidation and glucose utilization in response to PPARδ activation [38,85]. Anaerobic glycolysis via PPARδ upregulation is an advantageous metabolic pathway to sustain forced KC proliferation because it is a substantial source of ATP, which does not promote oxidative stress, in contrast to mitochondrial metabolism. The side effect of PPARδ upregulation might be the consumption, via ACOX1, of structural lipids, i.e., C24 and C26 fatty acids and ceramides destined to the stratum corneum, thus further compromising the epidermal inside-out barrier. Thus, upregulation of the PPARδ pathway in atopic and psoriatic lesions might be a double-edged sword, by sustaining KC proliferation without worsening oxidative stress but, at the same time, changing the composition of the lipid bilayer in the stratum corneum, resulting in less efficient barrier function. Thus, antagonizing PPARδ to correct metabolic abnormalities in lesional atopic dermatitis and psoriasis plaques might be a new and effective therapeutic strategy to reduce both epidermal hyperplasia and consumption of structural lipids of the stratum corneum lipid matrix.

## 4. PPARδ as a Therapeutic Target in Atopic Dermatitis and Psoriasis

To date, the therapeutic effects of PPARδ targeting in atopic dermatitis and psoriasis remain underinvestigated. Intriguingly, both PPARδ ligands and antagonists have been proven to dampen skin inflammation. Antagonism of PPARδ by topical application of GSK0660 in transgenic mice expressing Cyp1A1-driven expression of human *PPARD* in KCs and topically treated with the PPARδ agonist GW501516 (mouse model of psoriasis) reduced epidermal thickness, dermal inflammatory infiltrates with CD4^+^ and CD8^+^ T lymphocytes and expression of *Il1b* and *Lce3e* but failed to inhibit the expression of *Hb-egf* [100]. However, because the half-life of GSK0660 is only 90 min, this might be a limiting factor for its use as a therapeutic. Consequently, topical treatment with an irreversible PPARδ antagonist would be more appropriate to alleviate psoriasis symptoms. Indeed, a single topical treatment with GSK3787, which covalently binds and permanently inactivates PPARδ showed similar therapeutic efficacy as several topical applications with GSK0660 in mice with psoriasis-like skin inflammation [64,100]. Moreover, GSK3787 reduced the expression of *Il17*, *Il23a*, *Il22* and *Il1b* in these mice [64]. On the other hand, the activation of PPARδ with tetradecylthioacetic acid (TTA) also showed beneficial effects in psoriasis. In a small pilot study, topical treatment of psoriatic plaques with 0.5% TTA reduced the Psoriasis Area and Severity Index (PASI) and skin scaling and inflammation [101]. However, TTA can activate all PPAR isoforms at high doses [60]. Thus, the beneficial effects of TTA are likely the net result of the combined activation of all PPAR isoforms or a direct effect of the molecule. In a mouse model of dermatitis (i.e., mice topically treated with oxazolone, a chemical inducing Th2-predominant inflammation in mouse skin), topical application of GW1514, a PPARδ agonist, reduced epidermal hyperplasia, KC proliferation, transepidermal water loss, skin surface pH, skin infiltration by eosinophils and mast cells, and serum CCL17 [102]. However, it remains to be determined whether these effects are PPARδ-dependent. Topical treatment with GW1514 did not reduce serum IgE levels in oxazolone-treated mice [102], suggesting that this molecule does not reach the blood circulation after topical application. Thus, given the role of PPARδ in psoriasis and atopic dermatitis, PPARδ antagonism, rather than activation, might be the preferred therapeutic approach to treat both diseases. This does not mean that PPARδ ligands would be less advantageous therapeutic options; however, they should be mainly employed for their direct, i.e., PPAR-independent, beneficial effects.

Excessive oxidative stress overtaking the cellular antioxidant response is involved in tumorigenic processes, inflammation and skin aging. Accordingly, both psoriasis and atopic dermatitis are associated with oxidative stress [47,103,104,105]. The role of PPARδ in the antioxidant response is equivocal. Activation of PPARδ with GW501516 or other agonists has been shown to downregulate the mRNA and protein levels of NF-E2–related factor 2 (NRF2), a master transcription factor controlling the expression of key proteins involved in the cellular detoxification of reactive oxygen species (ROS) [106,107]. In contrast, PPARδ antagonism has been shown to promote the antioxidant response via upregulation of *Nrf2* [88] and to decrease the production of ROS in mitochondria [99]. In line with this, loss of PPARδ in intestinal fibroblasts delayed tumorigenesis, induced NRF2 and reduced oxidative stress [108]. The β-oxidation of very-long-chain fatty acids via ACOX1 produces hydrogen peroxide. In lesional atopic dermatitis and psoriasis, the marked increase in ACOX1 might outstrip the detoxification ability of the cellular antioxidant response and contribute to the epidermal oxidative stress observed in both diseases. Thus, overall, PPARδ might promote oxidative stress in the epidermis. Specifically, PPARδ might promote hydrogen peroxide release by peroxisomes (via ACOX1 activity) and, at the same time, dampen mitochondrial function and, in turn, the production of mitochondria-derived ROS. However, in non-skin cells, PPARδ ligands have been shown to prevent endoplasmic reticulum stress, downregulate NOX4 and reduce ROS production and subsequent inflammation [107,109]. Thus, we can speculate that PPARδ might exert both pro- and antioxidant functions as reported for other transcription factors [46], depending on pathophysiological context, cell type and organelle. Here again PPAR-independent antioxidant effects of PPARδ ligands might be envisaged. Unfortunately, the role of PPARδ in the oxidative response in KCs has never been investigated; PPARδ antagonism might have a potent antioxidant effect via mechanisms that remain to be identified.

Topical treatment with PPARδ agonists or antagonists should be critically evaluated because data on the role of PPARδ in cancer is controversial [19,85,110]. PPARδ has been shown to inhibit non-melanoma skin cancer by enhancing KC terminal differentiation and senescence, blocking KCs in the G2/M phase of the cell cycle, and inhibiting endoplasmic reticulum stress and specific inflammatory pathways [111,112,113]. However, PPARδ has also been shown to promote KC proliferation via HB-EGF and to contribute to epidermal hyperplasia [38,85]. Moreover, PPARδ can interact with β-catenin, a key mediator in the regulation of the Wnt pathway, which is involved in multiple cellular functions such as embryogenesis and tumorigenesis [114,115]. The overexpression of cytosolic phospholipase A2α (cPLA2α) promotes the binding of PPARδ to β-catenin and, in turn, the binding of the complex to the T-cell factor/lymphoid enhancer factor (TCF/LEF) response element [114,115]. cPLA2α is the rate-limiting enzyme which releases arachidonic acid from membrane phospholipids and, thus, playing a central role in the production of bioactive eicosanoids (including prostaglandins and leukotrienes), some of those are endogenous PPARδ ligands [116]. Thus, activation of PPARδ with endogenous ligands such as arachidonic acid or its derivatives may control cell fate (differentiation vs. proliferation) and malignant cell transformation. It has recently been shown that the PPARδ-β-catenin complex favors the formation of chromatin loops that regulate the transcription of vascular endothelial growth factor A (*VEGFA*), a regulator of angiogenesis during tumorigenesis. Activation of PPARδ via ligand binding releases the loop, which favors the transcription of *VEGFA* [115], and might sustain cancer growth. Furthermore, increased FABP5 is associated with various cancers including skin cancer, by promoting the activation of PPARδ and the upregulation of its oncogenic target genes [19]. It is possible that specific endogenous PPARδ ligands produced during tumorigenic transformation of cells skew PPARδ toward pro-oncogenic functions. The importance of the nature of ligands in driving PPARδ-mediated cellular responses is emphasized by work demonstrating the anti-apoptotic effects of PPARδ after activation with retinoic acid, which was shuttled to KC nuclei by FABP5 [85]. In tumors, this might help cancer cells escape apoptosis. Thus, activation of PPARδ in KCs by specific endogenous ligands might promote tumorigenesis by upregulating oncogenic genes, increasing oxidative stress and favoring a metabolic shift toward anaerobic glycolysis, which might promote non-melanoma skin cancer. Alternatively, competition of synthetic ligands with endogenous ligands to bind to PPARδ might positively intercede in the cellular response in tumors. Although PPARδ is expressed in melanocytes, its role in this cell type has never been investigated, which seems a missed opportunity since ligand-mediated PPARδ activation might protect against melanoma [117,118]. Thus, the role of PPARδ in skin tumorigenesis remains controversial, and the opposing views might owe to the use of different cancer cell lines, patient tissues, cancer staging and progression [7].

An important parameter for the topical utilization of drugs targeting PPARδ to alleviate atopic dermatitis and psoriasis is their transdermal absorption and ability to passage into the bloodstream. Indeed, systemic administration of GW501516 in a mouse model of wound healing showed that PPARδ activation promotes angiogenesis and upregulates matrix metalloproteinase 9 (MMP9) in wounded skin [85,119]. MMP9 is involved in many biological processes and plays roles in tumor progression and invasion, angiogenesis, and determining the composition of the tumor microenvironment [120].

Thus, the competition between endogenous and synthetic ligands/antagonists in a defined pathophysiological context (e.g., inflammation, precancer) might determine the therapeutic versus detrimental outcome of PPARδ targeting. This might also depend on the expression of corepressors/coactivators and other transcription factors engaged in PPARδ transrepression. Due to the therapeutic potential of PPARδ targeting in atopic dermatitis and psoriasis, further studies are necessary to elucidate in depth the role of PPARδ in the skin in various pathophysiological contexts and cell types (e.g., melanocytes) as well as the complex interplay between PPARδ and other transcription factors. Moreover, it is likely that synthetic ligands do not entirely activate PPARδ and that a small fraction of PPARδ remains activated by FABP5-bound endogenous ligands, leading to synergetic or contradictory signals, within cells. This aspect of PPARδ targeting is completely unexplored.

## 5. Conclusions

**Figure 1:**
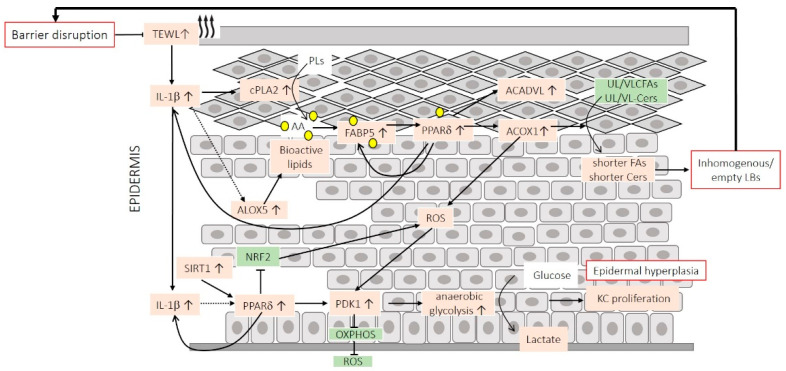
Potential role of PPARδ in keratinocytes in lesional atopic dermatitis and psoriasis: Epidermal barrier impairment, likely originating from (epi)genetic abnormalities, enhances trans-epidermal water loss (TEWL) and the production of IL-1β in granular keratinocytes (KCs), which upregulates cPLA_2_ involved in the cleavage of membrane phospholipids (PLs) and the release of arachidonic acid (AA). AA and its metabolites, produced by oxidation via ALOX5 into bioactive lipids, are shuttled to the nucleus by FABP5 to activate PPARδ, which, in turn, increases the expression of ACOX1 and ACADVL. Increased ACOX1 consumes ultra- and very-long-chain fatty acids (UL/VLCFAs) and ceramides (Cers), resulting in the improper embedding of stratum corneum lipids into lamellar bodies (LBs), which weakens the efficacy of the stratum corneum barrier, hence perpetuating epidermal barrier impairment. Overactivity of ACOX1 produces excessive hydrogen peroxide, which might signal within granular KCs as well as through all the epidermal layers to cause oxidative stress and metabolic changes. This might be amplified by the downregulation of NRF2 by endogenous ligand-bound PPARδ. In the basal layers, IL-1β, produced either locally or in granular KCs, and SIRT1, which is produced in the lower epidermis, contribute to the activation of PPARδ via unidentified mechanisms. This results in the upregulation of PDK1 and the shift toward anaerobic glycolysis, which circumvents mitochondrial function, including the production of mitochondrial ROS. Anaerobic glycolysis sustains KC hyperproliferation via rapid ATP production.

Between the years 2000 and 2010, PPARs were thoroughly studied in various organs including skin, but then, enthusiasm significantly waned. Moreover, much of the initial research was focused on PPARα and PPARγ, leaving large gaps in our knowledge of the role of PPARδ in the skin and especially in KCs. Thus, it remains unknown how PPARδ controls KC metabolism or the inflammatory response or the oxidative stress response. Furthermore, PPARδ crosstalk with other receptors such as VDR accentuates its importance in epidermal homeostasis. Therefore, in light of its clear involvement in KC proliferation, differentiation, metabolism, oxidative stress and the inflammatory response (Figure 1), renewed effort should be directed at both basic research and therapeutic strategies targeting PPARδ, including potential local and systemic side effects in psoriasis and atopic dermatitis.

## Figures and Tables

**Table 1:** PPARδ potential endogenous ligands.

Compounds	Weak Ligands	Ligands
ω3-PUFA	α-Linolenic acid C18:3	EPA C20:5
	γ-Linolenic acid C18:3	
	Dihomo-γ-linolenic acid	
	DHA C22:6	
ω6-PUFA	Linoleic acid C18:2	
	Arachidonic acid C20:4	
ω9-MUFA	Palmitoleic acid C16:1	Oleic acid C18:1
	Elaidic acid C18:1	
	Erucic acid C22:1	
	Nervonic acid C24:1	
Saturated fatty acids	Myristic acid C14:0	Arachidic acid C20:0
	Palmitic acid C16:0	
	Stearic acid C18:0	
	Behenic acid C22:0	
Eicosanoids	5-HpETE	5(S)-HETE
	8(S)-HETE	15(R)HpETE
	15(S)HpETE	15(R)-HETE
	15(S)-HETE	12-HETE
	12-HpETE	LTB4
	LTA4	LTC4
	9(R)-HODE	9(S)-HODE
	12-HpODE	5,6-diHETE
	13(S)-HODE	
	5,15-di-HpETE	
Prostaglandins	PGA2	PGF1α
	PGB1	
	PGB2	
	PGD1	
	PGD2	
	PGD3	
	PGF2α	
	PGF3α	
	PGI2	
Lipoxins		LXA4
4-Hydroxyalkenals	4-HDDE	

Adapted from [8]. DHA: docosahexaenoic acid; EPA: eicosapentaenoic acid; 4-HDDE; 4-hydroxydodecadienal; HETE: hydroxyeicosatetraenoic acid; HODE: hydroxyoctadecadienoic acid; LT: leukotriene; LX: lipoxin; PG: prostaglandin.

**Table 2:** PPARδ target genes and associated pathways in keratinocytes.

	Upregulated	Downregulated
Fatty acid metabolism	FABP5	LASS6
	FABP7	GPD1L
	ACADVL	PRKAB2
	ACOX1	CHPT1
	CD36	
	ALOX12B	
	LDLR	
	PLA2G3	
	ECHB	
	OACT5	
	BDH1	
	GDPD3	
	CRABP2	
	GM2A	
Cholesterol metabolism	HMGCS1	
	HMGCR	
	MVD	
	CYP51	
	SQLE	
	FDPS	
	LSS	
	FDFT1	
	DHC7	
KC proliferation	HB-EGF	EGFR
		EPS15
		EPS8
		MCC
		RBL2
		CCNG1
		DUSP3
		PDGFRA
		PDGFC
		CDKN1C
KC differentiation	INV	DCN
	TGM1	KRT15
	TGM3	DUSP3
	S100A8	
	S100A9	
	S100A16	
	KRT6B	
	KRT16	
	KRT17	
	KRT75	
	SPRR1B	
	CNFN	
	EHF	
KC apoptosis	CIDEA	
Inflammation	MMP9	TGFBR2
	IL1F9	TGFBR3
	IL1F5	LIFR
	IL1B	IL1R1
	IL1F6	
	IL1F8	
	ILA	
	IL1RA	
	IL18	
	IL17	
	IL23A	
	IL22	
	STAT3	
Glucose metabolism	PDK1	PDK4
Oxidative stress	SOD2	
	CAT	
	ABCC3	
Other	HAS3	RBL2
	GGH	AXL
	UCK2	RHOC
	ATP10B	TTC3
	CCNB1	LFNG
	MAPK13	FXR1
	CCNB2	FBLN1
	GSPT1	GAB2
	XPC	
		PIK3IP1
Unknown	AKR1B1	SERINC1
	ATP12A	EID1
	ACPP	KLF6
	MAP4K4	RAI14
	MREG	MTCP1
	FGFBP1	REEP5
	ARL8B	NENF
	GAS7	
	CD81	
	CCDC50	
	TACC1	
		OSR2

ABCC3: ATP binding cassette subfamily C member 3; ACAD(V)L: (very) long-chain specific acyl-CoA dehydrogenase, mitochondrial; ACOX1: acyl-CoA oxidase 1; ACPP (ACP3): acid phosphatase 3; AKR1B1: aldo-keto reductase family 1 member B; ALOX: lipoxygenase; ATP10B: ATPase phospholipid transporting 10B; ATP12A: ATPase H+/K+ transporting non-gastric alpha2 subunit; ARL8B: ADP ribosylation factor like GTPase 8B; AXL: AXL receptor tyrosine kinase; BDH1: 3-hydroxybutyrate dehydrogenase 1; CAT: catalase; CCDC50: coiled-coil domain containing 50; CCN: cyclin; CD: cluster of differentiation; CDKN1C: cyclin dependent kinase inhibitor 1C; CHPT1: choline C phosphotransferase 1; CIDEA: cell death inducing DFFA like effector A; CNFN: cornifelin; CRABP2: cellular retinoic acid binding protein 2; CYP51: lanosterol 14α-demethylase; DCN: decorin; DHC7 (DNAH1): dynein axonemal heavy chain 1; DUSP3: dual specificity phosphatase 3; ECHB (HADHB): hydroxyacyl-CoA dehydrogenase trifunctional multienzyme complex subunit beta; EGFR: epidermal growth factor receptor; EHF: ETS homologous factor; EID1: EP300 interacting inhibitor of differentiation 1; EPS: epidermal growth factor receptor pathway substrate; FABP: fatty acid binding protein; FBLN1: fibulin 1; FDFT1: farnesyl-diphosphate farnesyltransferase 1; FDPS: farnesyl diphosphate synthase; FGFBP1: fibroblast growth factor binding protein 1; FXR1: FMR1 autosomal homolog 1; GAB2: GRB2 associated binding protein 2; GAS7: growth arrest specific 7; GDPD3: glycerophosphodiester phosphodiesterase domain containing 3; GGH: gamma-glutamyl hydrolase; GM2A: GM2 ganglioside activator; GPD1L: glycerol-3-phosphate dehydrogenase 1 like; GSPT1: G1 to S phase transition 1; HAS3: hyaluronan synthase 3; HB-EGF: heparin-binding EGF-like growth factor; HMGCR: 3-hydroxy-3-methylglutaryl-CoA reductase; HMGCS1: 3-hydroxy-3-methylglutaryl-CoA synthase 1; IL: interleukin; INV: involucrin; KLF6: kruppel like factor 6; KRT: keratin; LASS6 (CERS6): ceramide synthase 6; LDLR: low density lipoprotein receptor; LFNG: LFNG O-fucosylpeptide 3-beta-N-acetylglucosaminyltransferase; LIFR: LIF receptor subunit alpha; LSS: lanosterol synthase; MAP4K4: mitogen-activated protein kinase kinase kinase kinase 4; MAPK13: mitogen-activated protein kinase 13; MCC: MCC regulator of WNT signaling pathway; MMP9: matrix metalloproteinase 9; MREG: melanoregulin; MTCP1: mature T cell proliferation 1; MVD: mevalonate diphosphate decarboxylase; NENF: neudesin neurotrophic factor; OACT5 (LPCAT3): lysophosphatidylcholine acyltransferase 3; OSR2: odd-skipped related transcription factor 2; PDGFC: platelet derived growth factor C; PDGFRA: platelet derived growth factor receptor alpha; PDK: pyruvate dehydrogenase kinase; PIK3IP1: phosphoinositide-3-kinase interacting protein 1; PLA2G3: phospholipase A2 group III; PRKAB2: protein kinase AMP-activated non-catalytic subunit beta 2; RAI14: retinoic acid induced 14; RBL2: RB transcriptional corepressor like 2; REEP5: receptor accessory protein 5; RHOC: ras homolog family member C; S100A: S100 calcium-binding protein A; SERINC1: serine incorporator 1; SOD2: superoxide dismutase 2; SPRR1B: small proline rich protein 1B; SQLE: squalene epoxidase; STAT: signal transducer and activator of transcription; TACC1: transforming acidic coiled-coil containing protein 1; TGFBR: transforming growth factor beta receptor; TGM: transglutaminase; TTC3: tetratricopeptide repeat domain 3; UCK2: uridine-cytidine kinase 2; XPC: XPC complex subunit, DNA damage recognition and repair factor.

## Data Availability

Not applicable.

## Abbreviations

ACAA2: acetyl-CoA acyltransferase 2; ACAD(V)L: (very) long-chain specific acyl-CoA dehydrogenase, mitochondrial; ACOX1: acyl-CoA oxidase 1; ACSL: long-chain-fatty-acid—CoA ligase; ACSS: acyl-CoA synthetase short chain family member; ALOX: lipoxygenase; ANGPTL4: angiopoietin Like 4; AP-1: activator protein 1; aP2: adipocyte protein 2; ATP: adenosine triphosphate; BCL-6: B-cell lymphoma 6; cAMP: cyclic adenosine monophosphate; CAT: catalase; CCL: CC-chemokine ligand; CD: cluster of differentiation; C/EBP: CCAAT-enhancer-binding protein; cPLA2α: cytosolic phospholipase A2α; CPT1: carnitine palmitoyltransferase I; CXCL: C-X-C motif Chemokine Ligand; CYP1A1: Cytochrome P450, family 1, subfamily A, polypeptide 1; ECH1: enoyl-CoA hydratase 1; EGF: epidermal growth factor; EGFR: epidermal growth factor receptor; ETFB: electron-transfer-flavoprotein, beta subunit; ETFDH: electron transfer flavoprotein-ubiquinone oxidoreductase; FABP: fatty acid binding protein; FLG: filaggrin; HB-EGF: heparin-binding EGF-like growth factor; HDAC: histone deacetylase; HEE: human epidermal equivalent; HETE: hydroxyeicosatetraenoic acid; 4-HDDE: 4-hydroxydodecadienal; HLA: human leukocyte antigen; 4-HNE: 4-hydroxynonenal; HODE: hydroxyoctadecadienoic acid; IG: immunoglobulin; IL: interleukin; INV: involucrin; ISCA1: iron-sulfur cluster assembly 1; JNK: Jun amino-terminal kinases; KC: keratinocyte; LCE3e: late cornified envelope 3e; LRP5: LDL receptor related protein 5; LTB_4_: Leukotriene B_4_; MEKK1: mitogen-activated protein kinase kinase kinase 1; MMP9: matrix metalloproteinase 9; NCOA/SIRT: nuclear receptor coactivator; NF-AT: nuclear factor of activated T-cells; NF-κB: nuclear factor kappa-light-chain-enhancer of activated B cells; NLRC4: NLR family CARD domain containing 4 protein; NLRP3: nucleotide-binding oligomerization domain-like receptor (NOD)-like receptor family pyrin domain containing 3; NPC1L1: Niemann-Pick C1-like protein 1; NRF2: NF-E2–related factor 2; NOX4: nicotinamide adenine dinucleotide phosphate (NADPH) oxidase 4; OXPHOS: oxidative phosphorylation; PASI: psoriasis area and severity index; p38MAPK: p38 mitogen-activated protein kinase; PDH: pyruvate dehydrogenase; PDK: pyruvate dehydrogenase kinase; PG: prostaglandin; PHACTR1: phosphatase and actin regulator 1; PI3K: phosphoinositide-3-kinase; PK: protein kinase; PLIN2: perilipin 2; PPAR: peroxisome proliferator-activated receptor; ROS: reactive oxygen species; RXR: 9-cis-retinoic acid receptor; S100A: S100 calcium-binding protein A; SAPK: stress-activated protein kinase; SDS: sodium dodecyl sulfate; SENP2: small ubiquitin-like modifier (SUMO)-specific protease 2; SIRT1: silent mating type information regulation 2 homolog 1: SLC: solute carrier family; STAT: signal transducer and activator of transcription; TCF/LEF: T-cell factor/lymphoid enhancer factor; TGM1: transglutaminase 1; Th: T helper; TNF: tumor necrosis factor; TPA: phorbol 12-myristate 13-acetate; TR: T3-thyroid receptor; TTA: tetradecylthioacetic acid; UCP: uncoupling protein; VDR: vitamin D receptor; VEGFA: vascular endothelial growth factor A.
